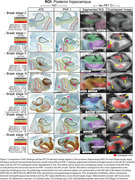# Tau‐PET overlap index correlates with neuropathology findings in early‐Braak stage

**DOI:** 10.1002/alz70856_106743

**Published:** 2026-01-08

**Authors:** Seokbeen Lim, Jeyeon Lee, Hoon‐Ki Min, Christina M. Moloney, Carly T. Mester, Sujala Ghatamaneni, Matthew L. Senjem, Aivi T. Nguyen, Jonathan Graff‐Radford, Christopher G Schwarz, Jeffrey L. Gunter, Kejal Kantarci, Brad F Boeve, Prashanthi Vemuri, David T. Jones, Clifford R. Jack, Ronald Petersen, Melissa E. Murray, Val J Lowe

**Affiliations:** ^1^ Mayo Clinic, Rochester, MN, USA; ^2^ Hanyang University, Seoul, Korea, Republic of (South); ^3^ Department of Radiology, Mayo Clinic, Rochester, MN, USA; ^4^ Mayo Clinic, Jacksonville, FL, USA; ^5^ Department of Laboratory Medicine and Pathology, Mayo Clinic, Rochester, MN, USA

## Abstract

**Background:**

The Overlap Index (OI) as previously published (Lee et al., 2022) is a reliable method for detecting tau accumulation using tau‐PET imaging (flortaucipir; AV1451). It identifies voxel‐wise standardized uptake value ratio (SUVr) elevations across serial scans. Although OI showed more sensitivity to tau signal elevation and longitudinal change than standard ROI measures, to our knowledge, the relationship of the tau PET overlap index to neuropathologic findings is still unclear. This study aims to investigate the potential of OI to detect NFTs of the neuropathologic Braak tangle stage, especially the early Braak tangle stage (I–IV).

**Method:**

This study included 57 participants from the Mayo Clinic Study of Aging or Mayo Alzheimer's Disease Research Center, all of whom underwent autopsy and at least two serial AV1451 tau‐PET scans and 3T T1‐weighted MRI within an average of 16 months. Tau‐PET SUVr images were normalized to cerebellar crus grey matter, and MR images were co‐registered to a single‐subject MRI mean. Tau‐PET scans were resampled into MRI space, and OI was calculated by applying intensity thresholds 1.3. Clusters with fewer than 20 contiguous voxels were excluded. An OI cutoff of 0.5 was used and an SUVr cutoff of 1.29. Postmortem immunohistochemistry (IHC) using phospho‐tau antibodies (AT8, PHF‐1) was performed on the amygdala, temporal cortex (middle and superior), and anterior/posterior hippocampus. Neuropathological Braak staging was determined based on AT8‐stained sections. We conducted visual comparison of tau‐PET overlap signal in these regions with IHC‐confirmed tau pathology across Braak tangle stages.

**Result:**

OI‐detected voxels corresponded to NFT distribution across Braak stages. In early Braak stages, standard SUVr‐based META‐ROI values remained negative, whereas OI showed positive signals, indicating greater sensitivity to early tau pathology (Figure 1). The spatial distribution of OI‐derived overlap regions aligned with high NFT burden, suggesting that OI captures early‐stage tau pathology more effectively than conventional SUVr methods.

**Conclusion:**

OI in serial tau‐PET imaging demonstrated better detection of early‐stage tau pathology than standard ROI‐based SUVr measures. The alignment between OI‐detected voxels and neuropathological tau burden supports its potential as an in vivo biomarker for detecting early neurofibrillary tangles.